# µ-Opioid Receptors Expressed by Intrinsically Photosensitive Retinal Ganglion Cells Contribute to Morphine-Induced Behavioral Sensitization

**DOI:** 10.3390/ijms232415870

**Published:** 2022-12-14

**Authors:** Nikolas Bergum, Casey-Tyler Berezin, Connie M. King, Jozsef Vigh

**Affiliations:** 1 Department of Biomedical Sciences, Colorado State University, Fort Collins, CO 80523, USA; 2 Cell and Molecular Biology Program, Colorado State University, Fort Collins, CO 80523, USA

**Keywords:** opioids, retina, μ-opioid receptor, photoentrainment, sleep/wake, addiction, behavioral sensitization, hypothermia

## Abstract

Opioid drugs are the most effective tools for treating moderate to severe pain. Despite their analgesic efficacy, long-term opioid use can lead to drug tolerance, addiction, and sleep/wake disturbances. While the link between opioids and sleep/wake problems is well-documented, the mechanism underlying opioid-related sleep/wake problems remains largely unresolved. Importantly, intrinsically photosensitive retinal ganglion cells (ipRGCs), the cells that transmit environmental light/dark information to the brain’s sleep/circadian centers to regulate sleep/wake behavior, express μ-opioid receptors (MORs). In this study, we explored the potential contribution of ipRGCs to opioid-related sleep/circadian disruptions. Using implanted telemetry transmitters, we measured changes in horizontal locomotor activity and body temperature in mice over the course of a chronic morphine paradigm. Mice lacking MORs expressed by ipRGCs (McKO) exhibited reduced morphine-induced behavioral activation/sensitization compared with control littermates with normal patterns of MOR expression. Contrastingly, mice lacking MORs globally (MKO) did not acquire morphine-induced locomotor activation/sensitization. Control mice also showed morphine-induced hypothermia in both the light and dark phases, while McKO littermates only exhibited morphine-induced hypothermia in the dark. Interestingly, only control animals appeared to acquire tolerance to morphine’s hypothermic effect. Morphine, however, did not acutely decrease the body temperature of MKO mice. These findings support the idea that MORs expressed by ipRGCs could contribute to opioid-related sleep/wake problems and thermoregulatory changes.

## 1. Introduction

Opioids remain some of the most powerful analgesics and are thus extensively used clinically for treating pain [1]. Therefore, it is important to carefully consider the dangerous side effects associated with their therapeutic use. While opioids are infamous for their addictive nature, their propensity to perturb sleep is often underappreciated. Both acute and chronic opioid administration have been shown to disrupt sleep/wake behaviors in patients [2,3,4]. Moreover, chronic opioid use has been associated with insomnia as well as daytime drowsiness [2,4,5,6]. These opioid-related sleep/wake problems often persist throughout chronic opioid treatment, which can exacerbate (existing) pain symptoms as well as the increase the risk for developing affective disorders [6,7,8,9]. Additionally, sleep disturbances have been cited as a primary contributor to opioid relapse and have been linked to increased suicidality [10,11]. Preclinical animal experiments have also shown that opioid administration disrupts healthy sleep/wake behaviors [12,13,14,15,16]. Opioid receptors are robustly expressed throughout the central nervous system (CNS), including regions of the brain implicated in sleep/wake regulation [12,17]. For instance, opioid receptors expressed by sleep-active neurons within the ventrolateral preoptic area (VLPO) of the hypothalamus [18], commonly referred to as the sleep switch [19], have been shown to mediate opioid-induced sleep/wake changes in rats [20]. Fentanyl has been shown to shift circadian rhythms in hamsters, presumably by modulating circadian pacemaker neuron signaling within the suprachiasmatic nucleus (SCN) of the hypothalamus [13,15,16]. Importantly, neurons within both the SCN and VLPO receive photic input from the retina [21]. 

Recent findings from our lab have demonstrated that a subset of ganglion cells in the mouse retina, the intrinsically photosensitive retina ganglion cells (ipRGCs), express μ-opioid receptors (MORs), the main molecular target for opioid drugs [22]. Indeed, MOR-selective agonist [D-Ala2, N-MePhe4, Gly-ol]-enkephalin (DAMGO) can effectively suppress light-evoked ipRGC firing [22]. These ipRGCs provide direct light information to the brain’s sleep/wake centers (e.g., SCN and VLPO), which synchronizes mammalian sleep/wake rhythms to environmental light conditions (i.e., photoentrainment) [23]. We have previously shown that endogenous opioid signaling in the retina modulates native sleep/wake behavior in mice via MORs expressed by ipRGCs [24]. Importantly, similar to opioid deposition in the human eye [25], morphine deposits in the mouse retina following systemic administration [26]. Thus, we wanted to test if MORs expressed by ipRGCs contribute to opioid-related sleep/wake disturbances in mice by telemetrically recording locomotor activity and body temperature upon acute and chronic intraperitoneal (i.p.) injections of morphine in freely moving mice.

Results revealed that acute and chronic morphine exposure differentially altered locomotor activity and body temperature of mice lacking MORs entirely (MKO, [27]) compared with those lacking MORs only in ipRGCs (McKO) and controls (mouse line containing a floxed version of the *Oprm1* gene, Mu^fl^, [28,29]). Acute morphine triggered transient hypothermia in control mice during both the subjective day and night that faded upon chronic administration. In McKO animals, transient hypothermia was observed upon both acute and chronic morphine administration in the subjective night, whereas in MKO mice, morphine had little effect on the body temperature. Both control and McKO mice exhibited increased locomotor movement following repeated i.p. morphine injections regardless of the injection time (i.e., behavioral sensitization [30]). Moreover, their behavioral activity became increasingly entrained to the morphine injections rather than the light schedule. Consistent with past findings, the MKO mice failed to acquire morphine-induced behavioral sensitization [31,32,33]. Nonetheless, while control and McKO mice both acquired morphine-induced behavioral sensitization, McKO mice exhibited reduced morphine-induced increases in locomotor activity compared with control littermates. Taken together, our data point to a previously unexplored retinal contribution to chronic opioid-induced disruption of photoentrainment as well as morphine-induced hypothermia.

## 2. Results

### 2.1. Prolonged Morphine Exposure Has Differential Effects on Locomotor Activity in Control, McKO, and MKO Mice

**Figure 1:**
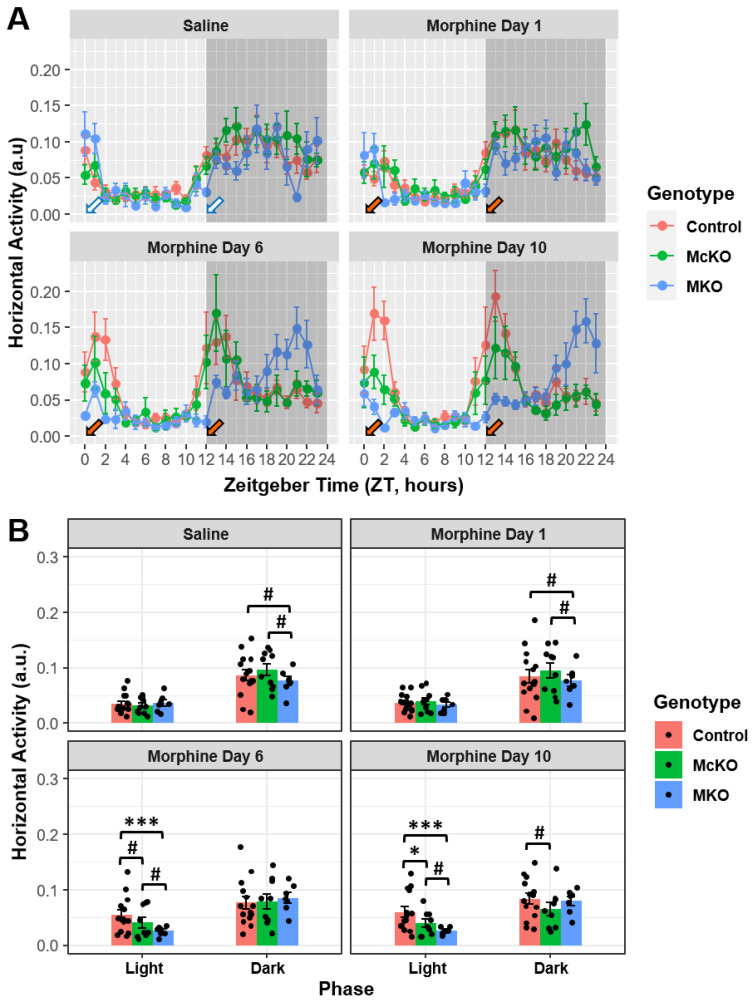
Prolonged but not acute morphine exposure differentially altered locomotor activity in Control, McKO, and MKO mice across light and dark phases. (**A**) Average behavioral activity plotted for Control (*n* = 14–15), McKO (*n* = 9–10), and MKO (*n* = 7) by hour (Zeitgeber Time) for different experimental days. Arrows indicate time of i.p. injection with either saline (white arrows) or 20 mg/kg of morphine (orange arrows). (**B**) Control and McKO animals exhibited increased horizontal activity compared with MKO animals. After prolonged morphine exposure, control mice exhibited increased locomotor activity compared with McKO littermates. Analysis performed using a three-way ANOVA (phase x genotype x experimental day) with a Holm–Bonferroni post hoc adjustment performed on all pairwise comparisons. (# *p* < 0.05, * *p* < 0.01, *** *p*<0.0001). Data presented as mean ± SEM.

Single-housed mice were implanted with mini-telemetry transmitters to record horizontal locomotor movement and body temperature within their respective cages every minute for the duration of the experiment. After recovery from transmitter surgeries, these mice were injected with saline twice daily at light onset (ZT 0) and light offset (ZT 12) for 6–7 days. Following the final saline injections on day 7, mice were injected with 20 mg/kg of morphine twice daily for 12 days [26,34]. To assess the effect of acute and prolonged morphine exposure on diurnal locomotor activity, we analyzed data from the final day of vehicle/saline treatment (Saline) as well as the data from the first day of morphine treatment (Morphine Day 1), the sixth day of morphine treatment (Morphine Day 6) and the tenth day of morphine treatment (Morphine Day 10). Notably, we chose to use day 10 as the last analysis day due to substantial data loss and attrition that occurred during days 11 and 12 of the morphine treatment paradigm. Additionally, we provided a range of animals for control and McKO groups, as subject attrition and data loss led to a decrease in usable data toward the end of the study. Analysis of these data revealed that each genotype responded differently to the chronic morphine treatment paradigm. Control (*n* = 15) and McKO (*n* = 10) littermates exhibited increased locomotor activity in the dark compared with aged-matched MKO (*n* = 7) mice during vehicle (Saline; Control vs. MKO: t(220) = 2.821, *p* = 0.0157; McKO vs. MKO: t(211) = 2.634, *p* = 0.0182) and acute morphine (Morphine Day 1; Control vs. MKO: t(220) = 2.580, *p* = 0.0316; McKO vs. MKO: t(211) = 2.433, *p* = 0.0316) treatment (Figure 1A,B). Interestingly, after subchronic morphine exposure (Morphine Day 6), there were no significant differences in dark phase horizontal activity across all genotypes (Figure 1A,B). Intriguingly, control mice (*n* = 14) showed increased locomotor activity in the dark compared with McKO littermates (*n* = 9) after chronic morphine exposure (Morphine Day 10; Control vs. McKO: t(214) = 2.927, *p* = 0.0114; Figure 1A,B). While there were no differences between different genotypes in light phase activity during saline and acute morphine treatment, subchronic (Morphine Day 6; Control vs. McKO: t(214) = 2.428, *p* = 0.0321; Control vs. MKO: t(219) = 4.426, *p* < 0.0001) and chronic morphine (Morphine Day 10; Control vs. McKO: t(214) = 3.206, *p* = 0.0031; Control vs. MKO: t(219) = 4.938, *p* < 0.0001) exposure increased control activity compared with McKO littermates and aged-matched MKO (*n* = 7) animals (Figure 1A,B). Additionally, McKO mice exhibited overall increases in dark phase locomotor activity following prolonged morphine administration (Morphine Day 6; McKO vs. MKO: t(210) = 2.276, *p* = 0.0321; Morphine Day 10; McKO vs. MKO: t(210) = 2.102, *p* = 0.0368; Figure 1A,B). 

Interestingly, chronic morphine (Saline vs. Morphine Day 10: t(206) = −3.099, *p* = 0.0133; Morphine Day 1 vs. Morphine Day 10: t(206) = −2.912, *p* = 0.0200) treatment, but not acute or subchronic morphine exposure, increased light phase activity in control mice compared with saline treatment; there was no impact on dark phase locomotor behavior across all experimental days (Figure S1A). Conversely, McKO mice exhibited decreased dark phase locomotor activity across the morphine treatment paradigm (Saline vs. Morphine Day 10: t(206) = 3.276, *p* = 0.0074; Morphine Day 1 vs. Morphine Day 10: t(206) = 3.076, *p* = 0.0119), while their light phase locomotor activity remained unchanged (Figure S1A). As a result, diurnal differences in horizontal activity ([Activity]dark phase—[Activity]light phase) for control and McKO mice were significantly decreased following subchronic (Saline vs. Morphine Day 6; Control: t(93.5) = 4.082, *p* = 0.0006; McKO: t(94) = 2.923, *p* = 0.0174) and chronic morphine (Saline vs. Morphine Day 10; Control: t(93.5) = 3.911, *p* = 0.0009; McKO: t(94) = 4.203, *p* = 0.0004) treatment when compared with vehicle-treated activity measures (Figure S1A). Meanwhile, morphine exposure did not seem to impact locomotor activity in MKO mice, regardless of treatment day (Figure S1A,B).

Upon subchronic and chronic morphine exposure, no significant differences were observed between McKO and MKO animals. However, McKO mice did appear to gradually decrease their dark phase activity throughout the treatment paradigm, while MKO mice did not exhibit any changes in average locomotor activity in either phase (Figure 1 and Figure S1). Furthermore, diurnal differences in overall horizonal activity decreased in McKO mice, but not MKO mice, following 6 and 10 days of morphine exposure (Figure S1). This suggests that these chronic morphine-induced decreases in activity across the paradigm were dependent on MOR-expressing cells outside of the retina (not ipRGCs). Perhaps more intriguingly, following subchronic and chronic morphine exposure, control mice exhibited increased activity relative to McKO littermates (Figure 1 and Figure S1). While both control and McKO mice showed reduced diurnal differences in horizontal locomotor activity, these differences resulted from progressive increases in control daytime activity throughout the morphine treatment regimen (Figure S1). These findings are consistent with past work showing that morphine-pellet-implanted rats exhibited a decrease in their diurnal activity amplitudes relative to those of controls [14]. Interestingly, while overall differences in light vs. dark activity decreased throughout protracted morphine treatment, the dark phase activity remained higher than that in the light phase across all experimental conditions (*p* < 0.01, Figure S2). This indicates that while morphine’s ability to alter mouse locomotor activity is robust, it is not enough to completely eliminate light’s ability to entrain the circadian rhythms of locomotor activity.

### 2.2. McKO Mice Display Diminished Morphine-Induced Behavioral Sensitization Compared with Control Littermates

**Figure 2:**
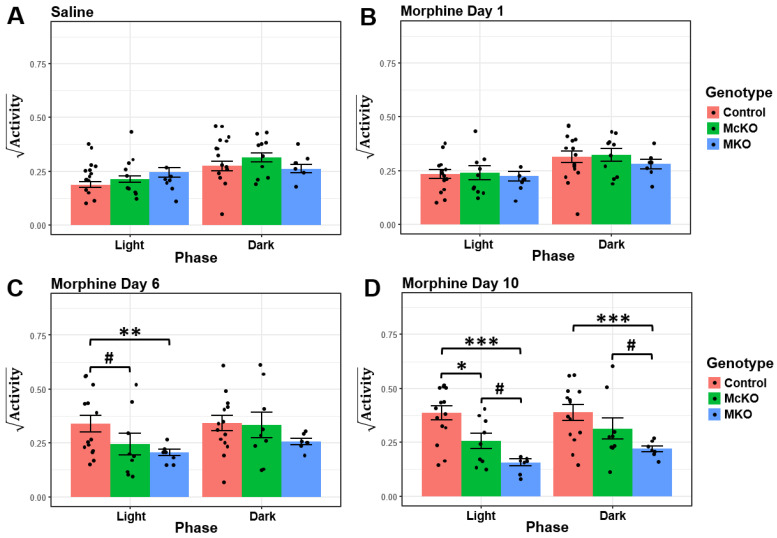
McKO mice exhibited reduced morphine-induced locomotor activation compared with control littermates following protracted morphine exposure. (**A**) Quantification of locomotor activity 1–2 h following saline injections at ZT0 and ZT12 for Control (*n* = 15), McKO (*n* = 10), and MKO (*n* = 7) mice. (**B**) Quantification of horizontal activity 1–2 h following initial 20 mg/kg i.p. morphine injections at ZT0 and ZT12 for Control (*n* = 15), McKO (*n* = 10), and MKO (*n* = 7) mice. (**C**) Quantification of horizontal activity 1–2 h following 20 mg/kg i.p. morphine injections at ZT0 and ZT12 for Control (*n* = 14), McKO (*n* = 9), and MKO (*n* = 7) mice on day 6 of the morphine treatment paradigm. (**D**) Quantification of horizontal activity 1–2 h following 20 mg/kg i.p. morphine injections at ZT0 and ZT12 for Control (*n* = 14), McKO (*n* = 9), and MKO (*n* = 7) mice on day 10 of the morphine treatment paradigm. Three-way ANOVA with a Holm–Bonferonni post hoc adjustment was performed on all pairwise comparisons (# *p* < 0.05, * *p* < 0.01, ** *p* < 0.001, *** *p* < 0.0001). Data presented as mean ± SEM.

The increase in control and McKO horizontal activity following morphine injections on day 6 and 10 was evident (Figure 1). This stereotyped increase in locomotor activity following repeated morphine exposure has been previously termed behavioral sensitization [30,35]. This behavioral phenomenon has been well established within the field, as rodents tend to increase their locomotor response over the course of chronic opioid paradigms [30,33,35]. To quantify morphine-induced increases in horizontal activity, we assessed horizontal locomotor activity during ZT 1, ZT 2, ZT 13, and ZT 14 (2–3 h) following a 20 mg/kg i.p. morphine injection at ZT 0 and ZT 12 (Figure 1A). Horizontal activity data were transformed (square root) to meet the normality and homogenous variance ANOVA assumptions. Notably, we excluded the first hour post-injection from our analysis, as locomotor depression has been observed in mice within the first ~30 min following systemic morphine administration, resulting in little total change in horizontal activity within the first hour [36,37,38]. When we compared differences in injection-induced horizontal locomotor activity across genotype, no significant differences were detected following saline and acute morphine treatment at ZT 0 and ZT 12 (Figure 2A,B). However, after six days of morphine exposure, control animals exhibited increased morphine-induced behavioral activity compared with both McKO littermates and MKO mice during the light phase (Control vs. McKO: t(221) = 2.341, *p* = 0.0403; Control vs. MKO: t(224) = 3.309, *p* = 0.0033; Figure 2C). This effect was amplified following 10 days of morphine treatment, as control animals retained their increased morphine-related locomotor activity compared with McKO and MKO mice in the light (Control vs. McKO: t(221) = 3.216, *p* = 0.0030; Control vs. MKO: t(224) = 5.527, *p* < 0.0001; Figure 2D). Control mice also showed increased behavioral activity compared with MKO mice in the dark following chronic morphine exposure (t(224) = 4.110, *p* = 0.0002; Figure 2D). Additionally, during Morphine Day 10, McKO mice exhibited increased post-injection horizontal activity compared with MKO animals in both the light (t(215) = 2.563, *p* = 0.0111) and the dark (t(215) = 2.446, *p* = 0.0305; Figure 2D). 

Interestingly, when we compared horizontal activity across experimental days within mice of the same genotype, only control animals acquired morphine-induced behavioral sensitization compared with vehicle injection days in both dark (Saline vs. Morphine Day 10: t(206) = −3.427, *p* = 0.0044) and light phases (Saline vs. Morphine Day 6: t(206) = −4.645, *p* < 0.0001; Saline vs. Morphine Day 10: t(206) = −6.108, *p* < 0.0001; Figure S3A,B). Meanwhile, McKO and MKO mice did not exhibit significant increases in post-injection locomotor activity over the course of the experiment (Figure S3C–F). While the role of MORs in the acquisition of behavioral sensitization is well-established [31,32,33], these data point to a potential contribution of MORs expressed by ipRGCs in morphine-induced locomotor sensitization.

### 2.3. Morphine Has Disparate Effects on Body Temperature across the Chronic Treatment Paradigm in Mice Differentially Expressing the MOR

**Figure 3:**
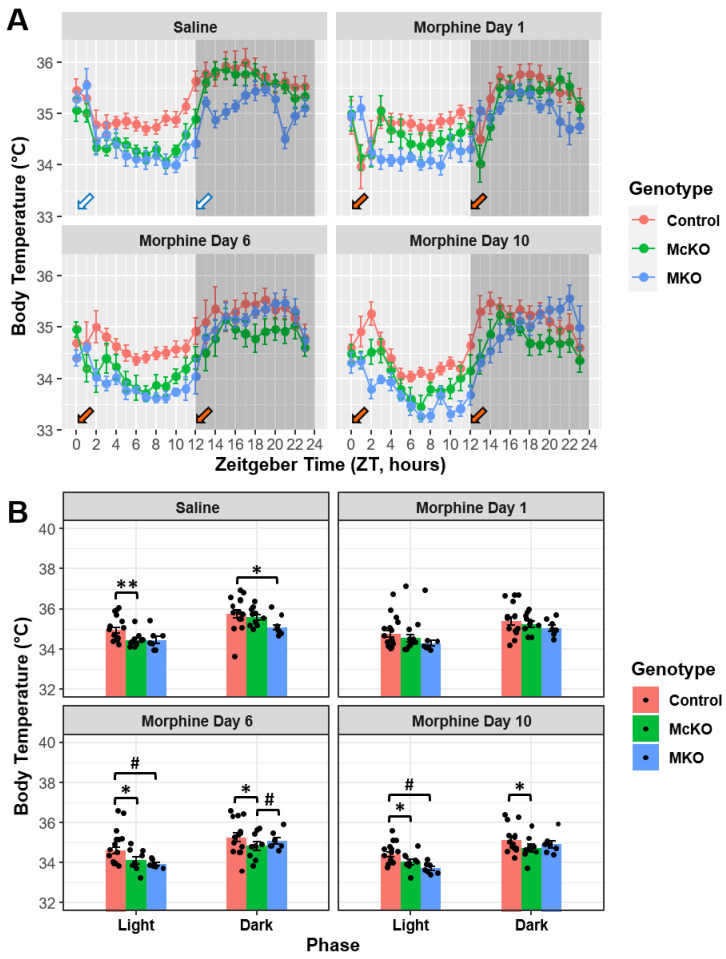
Morphine had differential effects on body temperature in control, McKO, and MKO mice across the morphine treatment paradigm. (**A**) Average body temperature plotted for control (*n* = 14–15), McKO (*n* = 9–10), and MKO (*n* = 7) mice by hour (Zeitgeber Time) for different experimental days. Arrows indicate time of i.p. injection with either saline (white arrows) or 20 mg/kg of morphine (orange arrows). (**B**) McKO mice had a lower mean body temperature than control littermates on all experimental days except for the first day of morphine treatment. Three-way ANOVA (phase × genotype × experimental day) with a Tukey post hoc adjustment was performed on all pairwise comparisons. (# *p* < 0.05, * *p* < 0.01, ** *p* < 0.001). Data presented as mean ± SEM.

Body temperature data were also obtained from implanted transmitters during the chronic morphine administration protocol described above. During the last day of vehicle treatment, control mice had higher average body temperature than McKO littermates in the light phase (Control vs. McKO: t(219) = 3.823, *p* = 0.0005); additionally, control animals showed increased body temperature compared with MKO animals in the dark phase following treatment with saline (Control vs. MKO: t(223) = 2.960, *p* = 0.0102; Figure 3A,B). Interestingly, no significant body temperature differences between the genotypes were detected upon acute morphine exposure (Morphine Day 1) (Figure 3A,B). However, differences between the genotypes returned following 6 and 10 days of morphine treatment, as control mice again had higher average body temperature than McKO littermates during both light (Morphine Day 6: t(217) = 3.659, *p* = 0.0010; Morphine Day 10: t(217) = 3.069, *p* = 0.0073) and dark (Morphine Day 6: t(217) = 3.338, *p* = 0.0028; Morphine Day 10: t(217) = 3.250, *p* = 0.0040) phases (Figure 3A,B). Additionally, control mice had higher body temperature compared with MKO mice in the light phase alone (t(223) = 2.768, *p* = 0.0122; Figure 3A,B), and McKO mice had decreased dark phase body temperature compared with MKO mice on Morphine Day 6 (t(212) = −2.494, *p* = 0.0268; Figure 3A,B). 

When we dissected the changes in body temperature across experimental days, it appeared that chronic morphine tended to decrease overall body temperature during both light and dark phases (except in the dark phase for MKO mice) (Figure S4A). In control mice, significant reductions in body temperature existed between Saline and Morphine Day 6 (light: t(206) = 2.543, *p* = 0.0499; dark: t(206) = 3.448, *p* = 0.0034) as well as Saline and Morphine Day 10 (light: t(206) = 3.780, *p* = 0.0012; dark: t(206) = 4.247, *p* = 0.0002) during both phases (Figure S4A). Similarly, McKO mice had reductions in dark phase body temperature between Saline and Morphine Days 6 (t(206) = 4.108, *p* = 0.0003) and 10 (t(206) = 4.664, *p* < 0.0001). Additionally, McKO mice exhibited significant differences between Morphine Day 1 and Morphine Day 10 in both the light (t(206) = 2.932, *p*= 0.0225) and dark phases (t(206) = 2.857, *p* = 0.0189; Figure S4A). MKO mice exhibited body temperature decreases when Saline was compared with Morphine Day 10 (t(205) = 3.285, *p* = 0.0072) and when Morphine Day 1 was compared with Day 10 (t(205) = 2.615, *p* = 0.0479) during the light phase, while MKO mice showed no such changes across experimental days in the dark phase (Figure S4A). Finally, it is important to note that nocturnal increases in overall body temperature were maintained for all experimental days across each genotype (*p* < 0.01; Figure S5).

### 2.4. Control Mice, but Not McKO or MKO Mice, Appear to Develop Tolerance to the Hypothermic Effects of Morphine during Chronic Administration

**Figure 4:**
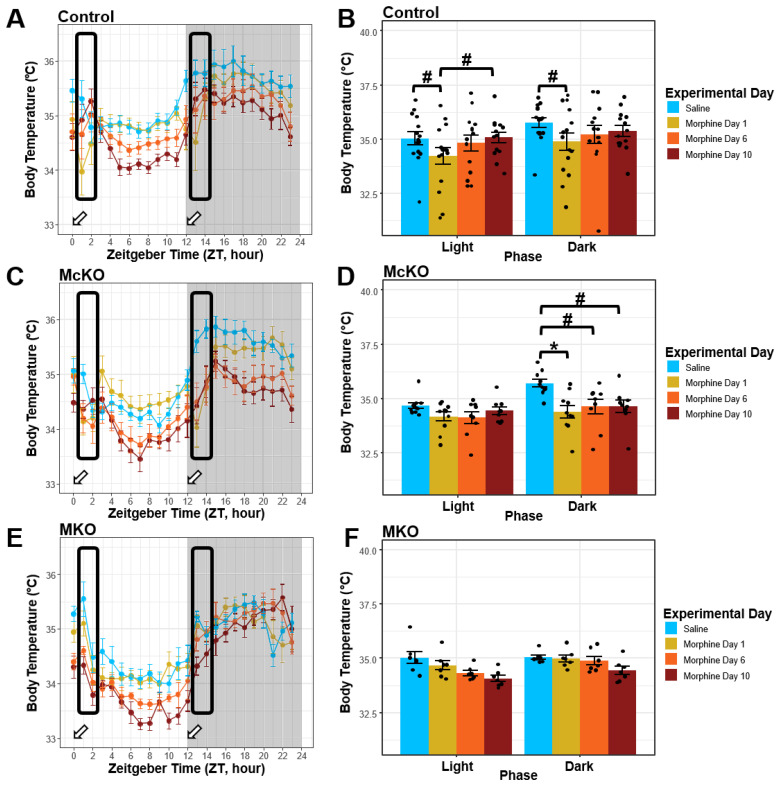
Control mice, but not McKO or MKO mice, developed tolerance to the hypothermic effects of morphine. (**A**,**B**) Control mice (*n* = 14–15) treated with chronic morphine exhibited morphine-induced hypothermia following acute but not chronic morphine exposure. Body temperature was measured 2–3 h following a 20 mg/kg i.p. morphine injection at ZT0 and ZT12. (**C**,**D**) McKO mice (*n* = 9–10) treated with morphine-induced hypothermia in the dark but not in the light. Body temperature was measured 2–3 h following a 20 mg/kg i.p. morphine injection at ZT0 and ZT12. (**E**,**F**) MKO mice (*n* = 7) exhibited minimal body temperature changes following both acute and chronic morphine administration. Body temperature was measured 2–3 h following a 20 mg/kg i.p. morphine injection at ZT0 and ZT12. Three-way ANOVA (phase × genotype × experimental day) with a Holm–Bonferroni post hoc adjustment was performed on all pairwise comparisons (# *p* < 0.05, * *p* < 0.01). Timing of injections indicated by the white arrows. Data presented as mean ± SEM.

Similar to the locomotor effects of morphine, the hypothermic response to high morphine doses has been shown to be biphasic, with a brief increase in body temperature followed by a longer-lasting period of hypothermia upon systemic administration [39]. Thus, to capture the peak of the acute morphine effect on body temperature, we looked at the window of time described above (2–3 h after i.p. morphine injections): ZT 1, ZT 2, ZT 13, and ZT 14. Analyses of this portion of the data revealed that control mice exhibited decreased body temperature in both light (Saline vs. Morphine Day 1: t(205) = 2.757, *p* = 0.0381) and dark (Saline vs. Morphine Day 1: t(205) = 2.944, *p* = 0.0217) phases following acute morphine exposure (Figure 4A,B). In the light phase, control mice showed a significant increase in body temperature between Morphine Day 1 and 10 (t(206) = −2.728, *p* = 0.0381); the body temperature was also increased (near saline baseline measurements) in the dark phase following prolonged morphine exposure (Saline vs. Morphine Day 10: t(206) = 1.405, *p* = 0.5629; Figure 4A,B). In the dark phase, McKO mice exhibited a similar reduction in body temperature compared to saline (t(205) = 3.667, *p*= 0.0019); however, this reduction persisted throughout the morphine treatment paradigm (Saline vs. Morphine Day 6: t(206) = 2.959, *p* = 0.0173; Saline vs. Morphine Day 10: t(206) = 2.955, *p* = 0.0173; Figure 4C,D). Importantly, no changes were detected in body temperature during the light phase (ZT 1 & ZT 2) in McKO animals (Figure 4C,D). Finally, MKO animals showed no significant changes in post-injection body temperature throughout the course of the treatment paradigm (Figure 4E,F). 

When we assessed differences across the three genotypes, we found that during the final saline day, control mice had higher body temperature than McKO littermates during the light phase (t(221) = 2.505, *p* = 0.0389; Figure S6A,B). Interestingly, on the first day of morphine administration, control mice had higher body temperature compared with McKO mice during the dark phase (t(221) = 2.926, *p* = 0.0114; Figure S6A,B). After six days of systemic morphine exposure, control mice retained their high body temperature compared with McKO littermates in the light phase (t(218) = 3.326, *p* = 0.0031) while also exhibiting increased core temperature in the dark phase (t(218) = 3.002, *p* = 0.0090; Figure S6A,B). Lastly, on Morphine Day 10, control mice exhibited higher body temperatures than both McKO and MKO mice after morphine injection at both light (Control vs. McKO: t(218) = 3.170, *p* = 0.0052; Control vs. MKO: t(224) = 2.990, *p* = 0.0062) and dark (Control vs. McKO: t(218) = 3.429, *p* = 0.0022; Control vs. MKO: t(224) = 2.822, *p* = 0.0104) phases. These data suggest not only that MORs are required for the hypothermic effects of high doses of morphine but also that MORs specifically expressed by ipRGCs play a modulatory role in tolerance development to morphine-induced hypothermia.

### 2.5. Morphine-Induced Behavioral Alterations in McKO Animals Likely Arise from MOR Expression Changes within the Retina

In order to determine whether the reported behavioral effects observed in McKO mice were due to alterations in MOR expression within the retina, we needed to validate that *Opn4* drove Cre recombinase (Cre) expression in ipRGCs and not in key regions within the brain. To accomplish this, we immunolabeled Cre in the hypothalamus (SCN and VLPO), striatum (caudate/putamen [CP]), and the paraventricular nucleus of the thalamus (PVT). We first labeled Cre in the retina of McKO mice crossed with *Opn4*-EGFP BAC-carrying mice (in which M1-M3 ipRGCs have green somas [22]). As shown in Figure S7A–C, we found strong somatic immunolabeling of Cre in McKO ipRGCs (26 cells from 2 animals, average cell volume = 213 μm^2^). Notably, we considered a cell a “true positive” if 5% of the cell’s volume was Cre+ and found that nearly 70% of ipRGCs were Cre+ (18/26 cells). Within cells that passed our 5% cutoff, the average Cre+ volume percentage in positive ipRGCs was 28.5% (maximum = 87.3%). Due to variable *Opn4* expression between ipRGC subtypes, only M1-M3 ipRGC subtypes can be identified in the *Opn4*-EGFP mouse [22]. Because we did not quantify Cre+ non-ipRGCs (Figure S7B,C), we likely underestimated the number of ipRGCs (especially in M4-M6 ipRGC subtypes) that were Cre+ in the McKO retina. 

To examine Cre expression in the brain, we first had to confirm that the antibody labeled Cre in the brain. To accomplish this, we sectioned the preoptic area of the hypothalamus from mice in which tdTomato was present in Cre-expressing galaninergic neurons (*Gal*-cre-tdTomato). As the preoptic area contains a high number of galanin-expressing cells [40], Cre immunolabeling in the *Gal*-cre-tdTomato brain also resulted in somatic labeling, albeit less robust than in the retina (Figure S7D–F). This was in strong contrast to the pattern of Cre immunostaining found in the McKO brain (Figure S8). In single optical sections from all analyzed brain regions, Cre appeared to be primarily localized to the outer surfaces of (i.e., surrounding) DAPI-labeled cells (Figure S8), suggesting that Cre immunoreactivity in the McKO brain primarily originated from Opn4+ ipRGC processes and not from neurons in the brain themselves. Nevertheless, as in the retina, we considered a cell a true positive if 5% of the cell’s volume was Cre+. We found that in the VLPO, 1.4% of cells were Cre+ (12/869; Figure S8A–C); in the SCN, 0.89% of cells were Cre+ (4/445); in the CP, 1.7% of cells were Cre+ (15/848 cells; Figure S8D–F); and in the PVT, 3.8% of cells were Cre+ (15/398; Figure S8G–I). Although we manually segmented most DAPI cell clusters (resulting in an average cell volume of 361 μm^2^; median = 341 μm^2^), the maximum cell volume (1480 μm^2^) indicates that some cell clusters remain, which may have led to artificially higher percentages of Cre+ cells. Given the low proportion of neurons that express Cre in the brains of McKO animals, their contributions to the behaviors described in this paper are likely small if not insignificant. 

## 3. Discussion

Synchronizing circadian activity rhythms to environmental light/dark cycles is essential for an organism’s ability to survive [23]. Mounting evidence points to synchronization of activity during specific light/dark phases to be crucial to optimal health of humans and rodents alike [23]. Results from the present study confirmed that chronic morphine exposure can drastically alter both circadian locomotor activity as well as body temperature rhythms [13,14,41]. Morphine primarily exerts its effect via MORs that are widely expressed throughout both the CNS as well as other body systems [42]. Thus, the observation that twice daily morphine exposure alters circadian behavioral/body temperature rhythms is not particularly surprising (Figure 1 and Figure S1). Indeed, this effect has been extensively demonstrated in both humans and rodents [4,10,11,12,13,14,16,41,43]. However, to our knowledge, no studies have examined the role of MORs expressed by retinal neurons in the context of opioid-related circadian dysregulation (Figure S5).

### 3.1. Systemic Morphine Affects Behavioral Activity via MORs Expressed by ipRGCs

Saline-injected mice lacking MORs (MKO) exhibited decreased horizontal activity compared with control/McKO aged-matched mice (Figure 1B). This finding is interesting, as it is consistent with the idea that increased levels of MOR-preferring β-endorphin at night potentially contribute to regulating behavioral activity [24,44]. On the other hand, it differs from previous findings from our lab that showed decreased dark phase activity in McKO mice compared with wildtype (C57BL6/J) animals [24]. This discrepancy in findings can be potentially attributed to two factors: (1) the use of littermate control animals (in this study) instead of wildtype mice as controls and (2) the stress of repeated injections utilized in the present study. Stress has been shown to alter circadian rhythms in both humans and rodents [45]. Thus, it is possible that the stress of repeated injections in the present study was enough to mask the decreases in nocturnal activity between McKO and control animals during the saline injection period. Interestingly, a pattern of decreased nocturnal behavior persisted after acute morphine exposure, which is consistent with the idea that the locomotor activation associated with morphine is acquired rather than innate [30]. 

Examining the activity traces across experimental days, we noticed that as mice proceeded through the chronic morphine paradigm, mouse activity became increasingly entrained to each morphine injection (Figure 1 and Figure S3). Not only was the activity entrained to these injections, but their horizontal locomotor activity appeared to increase over the course of the chronic morphine paradigm (Figure 1 and Figure S3). A similar effect has been reported in rats, wherein fentanyl-treated rats’ locomotion remained entrained to the drug infusion even after cessation of drug treatment [13]. Drug-induced hyperlocomotion (often referred to as behavioral activation) is a well-established phenomenon associated with rewarding properties of opioids and other drugs of abuse [30,46]. While this phenomenon was first described 50 years ago [47], our data are the first to suggest that ipRGCs contribute to this acquired drug-related behavior. 

To concentrate on the peak effect of morphine on horizontal activity, we specifically analyzed ZT 1, ZT 2, ZT 13, and ZT 14 (2–3 h) after daily injections at ZT 0 and ZT 12. Analysis of ZT 1, ZT 2, ZT 13, and ZT 14 revealed no differences between genotypes or experimental days during the light (ZT 1 & ZT 2) or dark (ZT 13 & ZT 14) phases post injection with vehicle and acute morphine (Figure 2 and Figure S3). However, after subchronic and chronic morphine exposure, differences began to emerge. Consistent with past findings [31,32,33,48], MKO mice did not acquire morphine-induced behavioral sensitization following chronic exposure and thus exhibited reduced post-injection locomotor activity compared with age-matched McKO and control animals (Figure 2 and Figure S3). This corroborates past experiments that have demonstrated the necessity of MORs for the acquisition of morphine-induced locomotor activation [31,32,33,48]. Surprisingly, MKO mice increased their locomotor activity between ZT 18 and ZT 23 following prolonged, but not acute, morphine treatment. This is even more interesting when we consider that after 6–12 h post injection, the morphine would have been systemically degraded [26]. This effect could result from a circadian activity shift mediated by morphine’s weak activity at the δ-opioid receptor (as δ-opioid receptor agonism has been shown to induce circadian phase shifts in rodents) [42,43]. Intriguingly, only control mice appeared to acquire morphine-induced behavioral sensitization (compared with saline baselines), while McKO littermates did not (Figure S3). Moreover, control animals showed increased post-injection horizontal activity following 6 and 10 days of chronic morphine treatment in the light phase but not the dark phase (Figure 2). This suggests that MORs expressed by ipRGCs might only play a role in morphine-induced behavioral activation during the light phase. Light has been shown to suppress locomotor activity in an ipRGC-dependent manner via both clock-dependent and clock-independent pathways [21,23,49]. Crucially, the MOR agonist DAMGO reduced light-evoked ipRGC firing via specific ionic currents [22], and morphine has been shown to accumulate in the mouse retina following chronic systemic injection [26]. Thus, morphine that accumulates in the retina could suppress the light-evoked ipRGC firing. MOR-mediated suppression of ipRGC activity could thus decrease light-mediated activation of sleep-promoting neurons in the brain’s sleep and circadian centers to increase activity in nocturnal animals [21,49]. While morphine certainly acts on other circadian, arousal, and reward centers within the brain to elicit behavioral sensitization/activation [12], to our knowledge, this study provides the first behavioral evidence that MORs expressed by ipRGCs are involved in morphine-related behavioral sensitization. 

### 3.2. Systemic Morphine Affects Thermoregulation via MORs Expressed by ipRGCs

Thermoregulation is an important physiological process that allows the body to properly function [50]. Circadian changes in body temperature in rodents are thought to correlate tightly with those of locomotor activity [51]. Morphine has been shown to differentially alter body temperature depending on the dose [39,52]. Importantly, activation of specific ipRGC subtypes has been linked to acute and circadian light-induced reductions in body temperature [49]. While circadian body temperature changes can sometimes precede circadian locomotor activity changes, typically, body temperature rhythms closely resemble circadian activity rhythms [51,53]. In mammals, both body temperature and locomotor activity are higher during the active phase and lower during the inactive phase [53]. While the two are not necessarily linked [51], locomotor activity can increase body temperature and thus mask the circadian body temperature rhythmicity [53]. Thus, it is important to consider that morphine-induced changes in body temperature could be affected by its locomotor effects. Diurnal variations in body temperature were conserved across all genotypes and experimental days (Figure S5). Specifically, control, McKO, and MKO mice all exhibited decreased average body temperature in the light phase across the morphine treatment regimen (Figure S4). Consistent with the activity data, extended morphine exposure reduced diurnal temperature amplitude in control and McKO mice but not MKO mice (Figure S4). Importantly, control and McKO mice only exhibited body temperature reductions in the dark phase following chronic morphine exposure (Figure S4). Notably, control mice had increased body temperature relative to McKO animals during subchronic and chronic morphine exposure (Figure 3). These data suggest that MORs expressed by ipRGCs play a role in the morphine-related body temperature changes in the dark phase but not the light phase. While these data are difficult to interpret, they indicate that MORs expressed by ipRGCs might play a role in morphine-induced alterations in body temperature regulation.

High doses of systemic morphine are known to induce robust body temperature decreases upon acute exposure [39,54]. Although many studies have examined morphine-induced hypothermia, the exact mechanism underlying this phenomenon remains unresolved [39]. Some rodent studies have suggested that the hypothermic effects of high-dose morphine are mediated by the κ-opioid receptor [55], which could explain the slight decrease in body temperature we observed on Morphine Day 10 (relative to saline) in the dark phase (Figure 4). However, the observation that the post-injection body temperature of MKO mice remained largely unaffected by vehicle/morphine treatment strongly implicates MORs in morphine-induced hypothermia. 

In the dark phase, control mice and McKO littermates exhibited significant reductions in post-injection body temperatures following acute morphine exposure (relative to saline controls) (Figure 4A,B). Interestingly, McKO littermates showed little change in post-injection body temperature throughout chronic morphine treatment (Figure 4B). Meanwhile, control mice exhibited reductions in morphine’s hypothermic effect over the course of chronic treatment (in both phases) (Figure 4A). Based on these data, Control animals appeared to develop tolerance to the hypothermic effects of morphine. While past studies have reported tolerance to the hypothermic effects of morphine [56], the mechanism by which this occurs remains largely unexplored. It is possible that chronic exposure reduces the hypothermic effect of morphine via MOR desensitization within the thermoregulatory pathway. However, upon concurrent examination of morphine’s effect on body temperature and locomotor behavior across the chronic treatment paradigm, it appears to us that morphine-induced increases in behavioral activity across the paradigm might be driving a gradual body temperature increase. In other words, the return of post-injection body temperature (near saline levels) could be due to the heat that is produced by morphine-induced behavioral activation. In support of this idea, both the hypothermic effects and the locomotor-stimulating effects of morphine have been linked to the mesolimbic dopamine pathway [30,57,58,59,60,61]. Moreover, opioid receptors in the preoptic area of the hypothalamus (POA) have been associated with both the wake-promoting and thermoregulatory properties of opioid drugs [18,20,55]. Taken together, these findings suggest that a convergent mechanism underlies both morphine-induced behavioral sensitization and the progressive reductions in morphine-induced hypothermia.

### 3.3. Strengths and Limitations

To gain a clearer understanding of morphine’s effect on circadian behavioral rhythms, we employed mini-telemetry devices to measure their horizontal locomotor activity and body temperature 24 h/day for the duration of each trial. While this method does require surgery, it provides a more complete picture of the animal’s locomotor behavior throughout the day when compared with traditional wheel-running experiments. First, wheel running is not able to record locomotor activity when mice are away from the wheel. Additionally, the inherent rewarding nature of wheel running [62] complicates the interpretation of the data when studying drugs of abuse such as morphine. To distill the morphine-specific effect on horizontal locomotor activity, we first treated the mice with saline for 5–6 days prior to morphine exposure. This helped the animals habituate to handling while also providing us with a behavioral baseline to compare to the drug treatment. Specifically, by comparing vehicle-injected animals to morphine-injected animals we controlled for any potential locomotor responses that the stress of handling and/or the i.p. injection itself might elicit. 

While the current study uncovered a potentially novel contributor to morphine-induced behavioral sensitization, this study was not without limitations. First, the use of the Control/McKO line yielded mice with at least a 75% C57BL/6J background, while the MKO mice were completely backcrossed to the C57BL/6J background. Importantly, the control line has been shown to exhibit normal morphine-induced antinociception and develop antinociceptive tolerance [28]. Moreover, this mouse line acquires opioid-induced behavioral activation/sensitization and self-administers opioids [29]. For the purposes of this study, the control/Mu^fl^ line functions normally with regard to opioid-related behavioral responses, and we thus felt it was acceptable to compare Control/McKO animals to age-matched MKO animals (despite minor differences in genetic background) [28]. 

Another potential limitation of our study was using the Cre-lox breeding strategy to generate McKOs where only ipRGCs are expected to lack MORs. Specifically, many *Opn4*-cre mouse lines have been shown to express Cre in retinal ganglion cells other than ipRGCs [63,64]. Consequently, other retinal neurons might lose functional MOR expression as a result of the cross between *Opn4*-cre mice and mice with a floxed version of the MOR. While *Opn4*-cre mice have been shown to label non-photosensitive retinal ganglion cells [64], it is unlikely that potentially removing MORs from these retinal ganglion cells would have any significant effect on light-mediated regulation of behavior. For example, in aging rats, loss of ipRGCs and no other retinal ganglion cells correlates well with circadian rhythm impairments [65]. Additionally, in a number of retinal diseases affected a variety of cell types, at the stages where alterations in circadian rhythms are detected, the misalignments have been positivity correlated with ipRGC loss [66]. This further substantiates the role of ipRGCs in mediating circadian photoentrainment over other types of retinal neurons that might express Cre and/or MORs. 

Interestingly, there is some evidence of *Opn4* expression outside of the mammalian retina. In humans, *Opn4* expression was detected in areas of the brain that express MORs. The most significant areas are the caudate, putamen, and nucleus accumbens [67]. Additionally, studies performed in C3H/HeNHsd rd/rd mice have indicated Opn4 expression in the hypothalamus and cerebellum [68]. Thus, *Opn4*-driven Cre expression could reduce functional MOR in specific brain regions involved in morphine-induced changes in locomotor activity and temperature regulation. However, we have previously shown that McKO animals do not exhibit significant differences in MOR expression compared to control [24]. Additionally, mouse studies in C57BL6 mice have shown that MOR-expressing cells in these regions (specifically the caudate, putamen, nucleus accumbens, and cerebellum) do not also express *Opn4* and vice versa [69]. This suggests that brain regions that are important for mediating morphine-induced locomotor activation would not have Cre-dependent decreases in MOR expression. Furthermore, our own validation of the McKO mouse line indicated that Cre expression was primarily restricted to cells in the retina (with minimal expression in the brain) (Figures S7 and S8). While experimental evidence points to the idea that Cre-dependent elimination of functional MOR expression occurs mostly within the retina, we acknowledge that the *Opn4*-cre mouse line could be functionally modulating MOR expression in a small subset of brain neurons that could affect morphine-driven behaviors. 

Animal handling complications limited this study to assessing the effect of morphine on body temperature and locomotor activity in freely moving animals (see Section 4). While we were not able to directly assess sleep/wake activity using EEG/EMG, telemetric locomotor activity monitoring has been shown to provide a reliable behavioral readout that correlates well with EEG/EMG-derived sleep/wake analysis in rodents [70]. Lastly, the present experiments were performed using exclusively male mice to avoid any behavioral complications associated with the estrus cycle. Future studies should examine the contribution of MORs expressed by ipRGCs to potential sex-dependent differences in the effect of opioids on circadian behaviors. These studies in female animals are crucial given mounting evidence of sex-related differences in the responsiveness to opioid drugs in rodents and humans [71,72,73].

## 4. Materials and Methods

### 4.1. Animals

*Oprm1^fl^/^fl^* (Mu^fl^) breeders (Jackson Labs strain #030074) were generously provided by Dr. Brigitte Kieffer (Douglas Research Center, McGill University). Mu^fl^ mice have exons two and three of the MOR (*Oprm1*) gene flanked by a *loxP* site and were maintained on a 50% C57BL/6J-50% 129Sv background for five generations before receipt [28]. Mice with Cre recombinase expressed upstream of the melanopsin (*Opn4*) promoter [Tg(*Opn4*-cre) SA9Gsat/Mmucd, #036544-UCD; *Opn4*-cre] were purchased from the Mutant Mouse Resource and Research Center (MMRRC) at the University of California, Davis. These *Opn4*-cre mice were backcrossed into a 100% C57BL/6J background prior to purchase and were maintained as hemizygotes (*Opn4*-cre +/−). MOR-conditional knockout (McKO) mice, in which ipRGCs lack functional MORs, were generated as described previously [24,74]. In brief, Mu^fl^ and *Opn4*-cre mice were crossed to obtain McKO mice with the floxed *Oprm1* gene on both alleles and *Opn4*-cre on one allele. Thus, the result of this cross produced 50% Mu^fl^/control: 50% McKO littermates. Notably, this Mu^fl^/McKO line was maintained on a background containing at least 75% C57BL/6J and 25% 129Sv. In all experiments, Mu^fl^ littermates of McKO mice were used as controls. Mice lacking functional MORs globally (B6.129S2-Oprm1tm1Kff/J; MKO) were purchased from Jackson Labs (strain #007559) [27]. MKO mice were backcrossed to C57BL/6J mice for at least 12 generations prior to purchase. 

For the validation of this McKO mouse line, we crossed McKO mice to *Opn4*-EGFP BAC-carrying mice (Tg(*Opn4*-EGFP)ND100Gsat/Mmucd strain, generated by the GENSAT project) [22]. To examine Cre expression within the brain, we used mice that had Cre recombinase under control of the *GAL* promoter (B6J.FVB(Cg)-Tg(*Gal*-cre)KI87Gsat/Mmucd; *Gal*-cre). These Gal-cre animals were crossed to Ai14 (Jackson Labs strain #007914) mice to express tdTomato following *GAL*-driven Cre-dependent recombination (*Gal*-cre-tdTomato). 

Adult male animals (8–26 weeks) were housed under a 12-h light:12-h dark cycle (LD) cycle, with lights on at 7:00 a.m. (ZT 0) and lights off at 7:00 p.m. (ZT 12). They were fed standard chow and water ad libitum. All animals used in these studies were handled in compliance with the Institutional Animal Care and Use Committees of Colorado State University (Protocol 18-8395A, 28 January 2019) and in accordance with the ARVO Statement for the Use of Animals in Ophthalmic and Vision Research.

### 4.2. Morphine Treatment

Mice were weighed and injected with doses of sterile saline or 20 mg/kg of morphine i.p. (Morphine sulfate salt pentahydrate, Sigma-Aldrich, Saint Louis, MO, USA; Product Number: M8777, dissolved in sterile saline). The dosage of 20 mg/kg i.p. morphine is commonly used to assess analgesic tolerance in mice [75]. Mice received two injections daily: one at light onset (ZT 0) and a second at light offset (ZT 12). Prior to morphine administration, mice were injected with sterile saline twice daily for 6–7 days (the corresponding figures show the last day). The chronic morphine treatment paradigm consisted of 12 days of twice daily 20 mg/kg of morphine (administered at ZT 0 and ZT 12), which was always proceeded by at least 5 days of twice daily saline injections on the same schedule [26]. Mice were weighed prior to surgery (preceding the injections) to ensure accurate dosages.

### 4.3. Surgery and Telemetry Recordings

Adult male Mu^fl^/control, McKO, and MKO mice were surgically implanted with mini-telemetry transmitters (HD-X02 or TA-F10, DSI) as previously described [24,76]. In brief, mice were deeply anesthetized with isoflurane (5%) and fitted with a nose cone for continuous isoflurane delivery (1–5%). Core body temperature and respiratory rate were monitored throughout surgery. Transmitters were subcutaneously inserted in the dorsal abdomen. For HD-X02transmitters, electromyograph (EMG) leads were inserted into the cheek muscles, and electroencephalogram (EEG) leads were placed into holes drilled into the skull (1.0 mm posterior to bregma, 1.0 mm left of midline; 2.0 mm posterior to bregma, 1.0 mm right of midline) and held in place with dental acrylic. After the incisions were sutured, mice received an injection of the non-steroidal anti-inflammatory drug (NSAID) OstiLox (3 mg/kg meloxicam, VetOne) and their status was recorded every 5 min for the first 30 min post-surgery. Mice continued to be monitored for three days in separate cages under a standard 12-h light:12-h dark cycle with lights on at 7:00 a.m. (ZT 0). After recovery, each cage was placed on a receiver (DSI), which relayed the telemetry signals to a computer for recording. It is important to note that handling the mice during our twice daily i.p. injection schedule resulted in loosening and/or breaking the EMG/EEG leads; therefore, our analysis was reduced to activity and body temperature measurements provided by both types of transmitters. The implanted transmitters recorded the horizontal movement/locomotor activity and body temperature of Mu^fl^/control, McKO, and MKO mice in their home cages [24]. Activity, measured in arbitrary units (a.u.), was recorded each minute. Mice were considered inactive when no horizontal movement was recorded during that minute. Similarly, body temperature measurements, measured in °C, were recorded each minute of the experimental trial for each animal.

### 4.4. Immunohistochemistry

Adult male mice were deeply anesthetized with pentobarbital sodium (Fatal-Plus) and then perfused with 10% sucrose in 0.1 M phosphate-buffered saline (PBS) followed by 4% PFA in PBS. The eyes were enucleated and post-fixed in 4% PFA at RT for 25 min (eyes were puncture-fixed for 20 min before the retinas were dissected out in PBS and returned to the fixative for an additional 5 min). Whole brains were removed and post-fixed in 4% PFA at 4 °C for at least 24 h and then cut into 100 μm sections on a Leica vibratome. Brain slices and whole-mount retinas were washed with PBS then placed in blocking solution (5% serum, 0.5% Triton-X-100 in 0.1 M PBS). Tissues were incubated in the rabbit anti-Cre antibody (1:500 Cell Signaling #15036) for approximately 24 h followed by the appropriate secondary antibody (Goat anti-Rabbit Alexa Fluor 647, ThermoFisher, Waltham, MA, USA). The tissues were stained with DAPI (GeneTex, Irvine, CA, USA) then mounted on Superfrost Plus microscope slides (Fisher Scientific, Hampton, NH, USA) in Vectashield Plus Antifade Mounting Medium (Vector Laboratories, Burlingame, CA, USA).

Tissues were imaged in 1 μm Z-stack increments on a LSM 900 confocal microscope (Carl Zeiss, Oberkochen, Germany) with a 40× oil-immersion objective. For all images, sequential scans at each wavelength were performed, and 4× averaging was performed to improve the signal-to-noise ratio. Automated image analysis was performed using Fiji (v1.53, [77]). In brief, after initial pre-processing (i.e., adjusting brightness and denoising), a median blur filter was applied to each channel, and the images were thresholded and converted to binary masks. The 3D Objects Counter plugin [78] was used to count DAPI+, GFP+, and/or Cre+ objects in each mask. The accuracy of GFP+ cell counting was manually verified, and most large (>700 µm^2^) DAPI+ cell clusters were manually watershed segmented using the 3D ROI Manager [79]. The “Measure” and “Colocalization” features of the 3D ROI Manager were used to calculate all objects’ numbers, volumes, and percent colocalization [79].

### 4.5. Data and Statistrical Analysis

Data are presented as mean ± standard error of the mean (SEM), as specified in figure captions. The data were analyzed using two-way (genotype × experimental day) or three-way (phase × genotype × experimental day) ANOVA. Some data were transformed to the square root scale to satisfy ANOVA assumptions. Data wrangling, visualizations, and analyses were performed using RStudio (version 4.0.0) with Holm–Bonferroni post hoc adjustments for all pairwise comparisons, and *p* < 0.05 was considered significant.

## 5. Conclusions

The results of this experiment established a potential link between MORs expressed by ipRGCs and opioid-induced locomotor sensitization. This finding is particularly surprising because most studies assessing the underlying mechanisms of morphine-induced behavioral activation/sensitization have focused on MOR expression within the mesolimbic dopamine reward circuit and not the photoentrainment pathway [29,30,35,58,60,73,80]. Behavioral sensitization and the positive-reinforcing effects of opioids have long been linked due to their concurrent temporal progression and overlapping neuroanatomical correlates [35,81,82]. Moreover, behavioral sensitization is considered to be a strong behavioral marker for addiction-associated behaviors [46,60]. Thus, the findings presented here, in conjunction with past work from our lab, not only suggest a retinal contribution to opioid-induced circadian disruptions but also point to ipRGC’s involvement in the mixed etiology of drug-related behaviors [22,24,26]. While we are not suggesting that ipRGCs are a primary mediator of these opioid-induced effects, this study presents ipRGCs a potentially novel target to ameliorate the effects of opioid-related sleep/wake disturbances.

## Data Availability

Publicly available datasets were analyzed in this study. This data can be found here: https://github.com/thebergular/ipRGC_MORs (accessed on 11 December 2022).

## Supplementary Materials

The supporting information can be downloaded at: https://www.mdpi.com/article/10.3390/ijms232415870/s1.
