# Supplementary material for: µ-Opioid Receptors Expressed by Intrinsically Photosensitive Retinal Ganglion Cells Contribute to Morphine-Induced Behavioral Sensitization
Source: Int J Mol Sci. 2022 Dec 14;23(24):15870. doi: 10.3390/ijms232415870 (PMC9781919; doi:10.3390/ijms232415870)
Supplement: Supplementary file 1 [file ijms-23-15870-s001.zip › ijms-1904917-supplementary.pdf]

International Journal of
Molecular Sciences

MDPI

Supplementary Materials:

Figure S1: Prolonged, but not acute morphine decreases diurnal variations in circadian behavioral activity;

Figure S2: All genotypes retain normal behavioral activity patterns throughout the course of the morphine treatment paradigm;

Figure S3: Protracted morphine exposure causes morphine-induced behavioral sensitization in Control, but not McKO or MKO mice;

Figure S4: Prolonged, but not acute morphine decreases body temperature in control, McKO and MKO mice;

Figure S5: All genotypes retain normal body temperature patterns throughout the course of the morphine treatment paradigm;

Figure S6: Mice that differentially express the MOR show body temperature changes in response to injections at different stages of a chronic morphine paradigm;

Figure S7: Positive controls show somatic Cre immunolabeling;

Figure S8: Cre immunolabeling in the McKO brain may originate from EGFP + ipRGCs.
