# Supplementary figures and images for: µ-Opioid Receptors Expressed by Intrinsically Photosensitive Retinal Ganglion Cells Contribute to Morphine-Induced Behavioral Sensitization
Source: Int J Mol Sci. 2022 Dec 14;23(24):15870. doi: 10.3390/ijms232415870 (PMC9781919; doi:10.3390/ijms232415870)

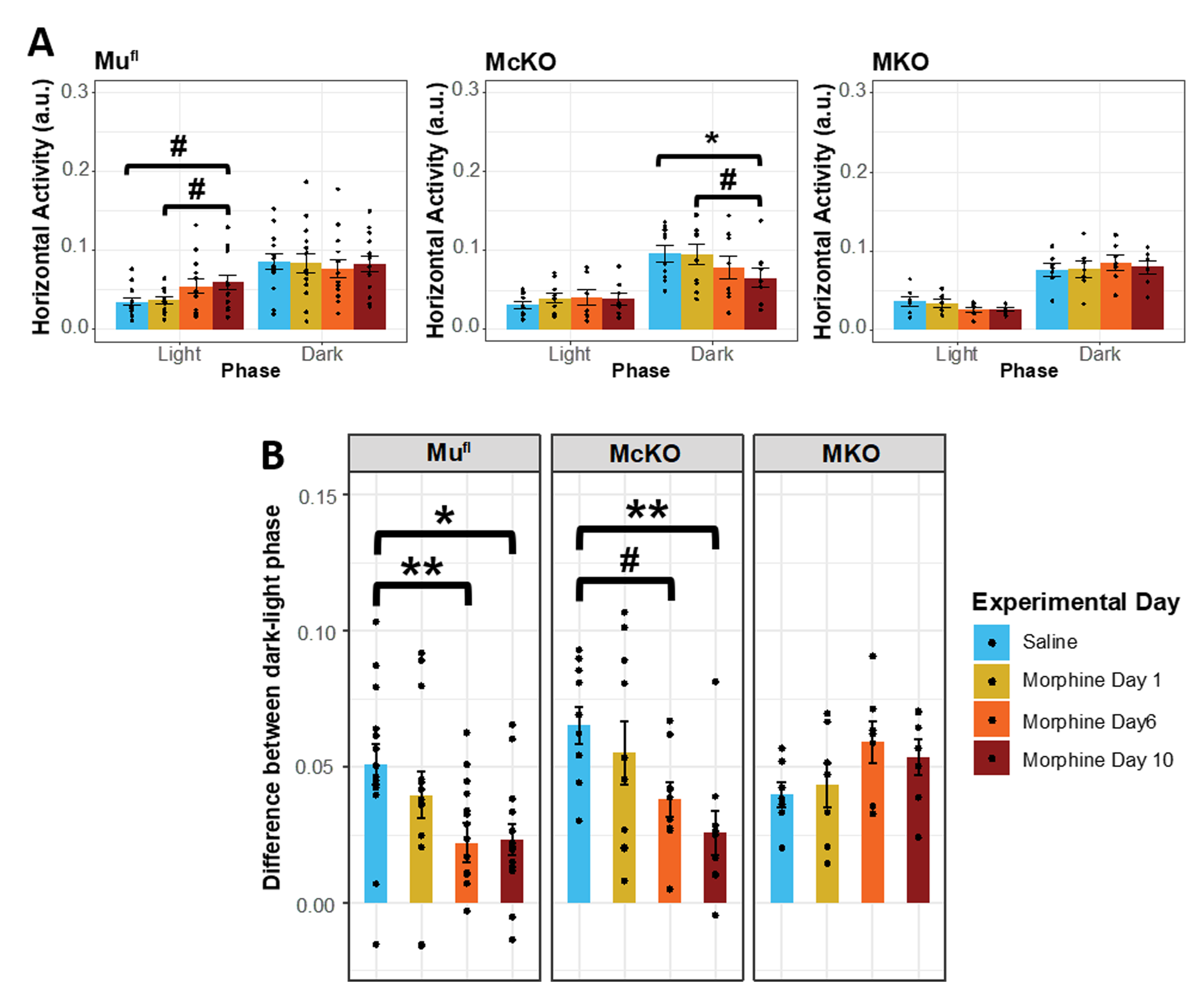

Supplement: Supplementary file 1 [file ijms-23-15870-s001.zip › FigureS1.tif]

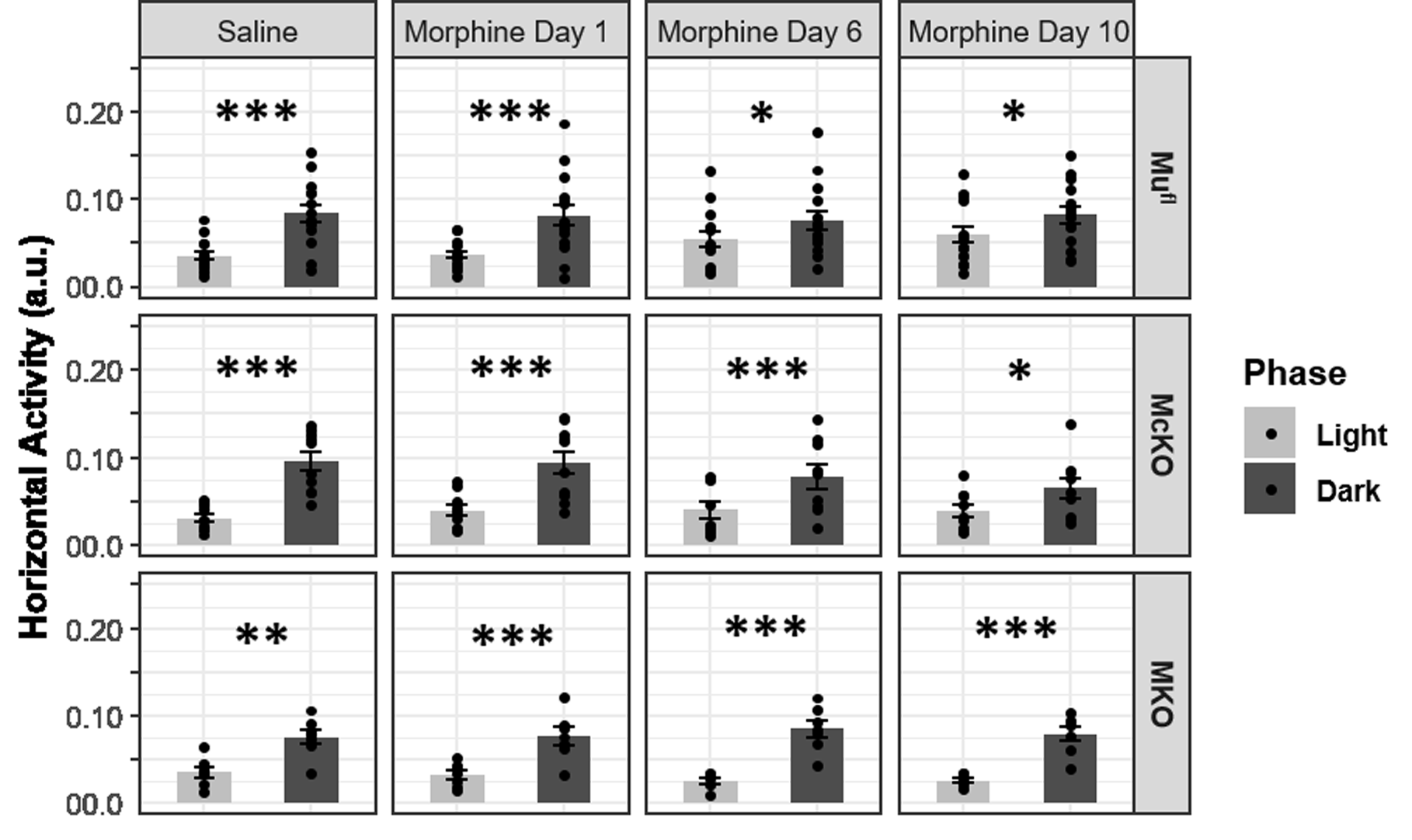

Supplement: Supplementary file 1 [file ijms-23-15870-s001.zip › FigureS2.tif]

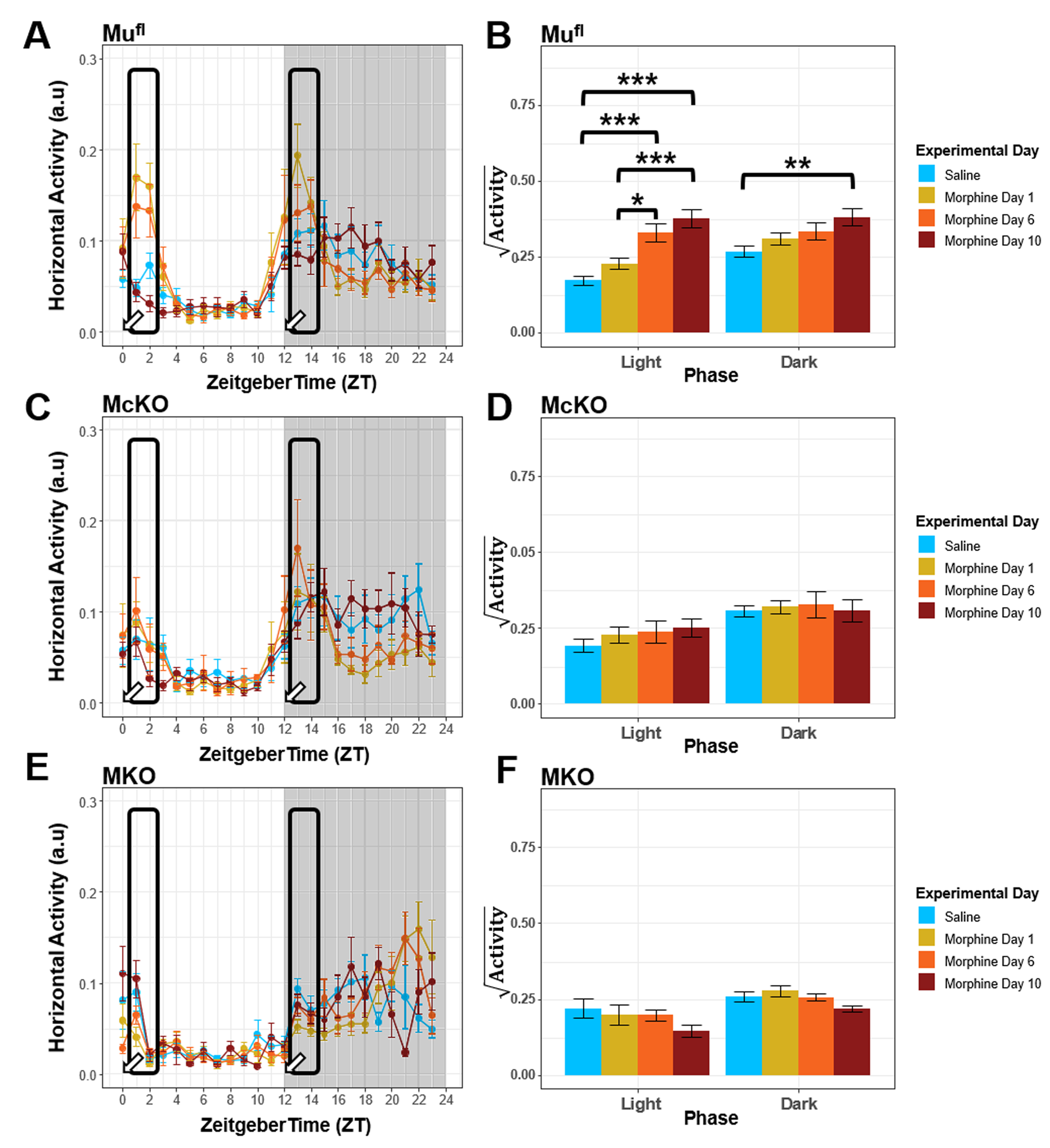

Supplement: Supplementary file 1 [file ijms-23-15870-s001.zip › FigureS3.tif]

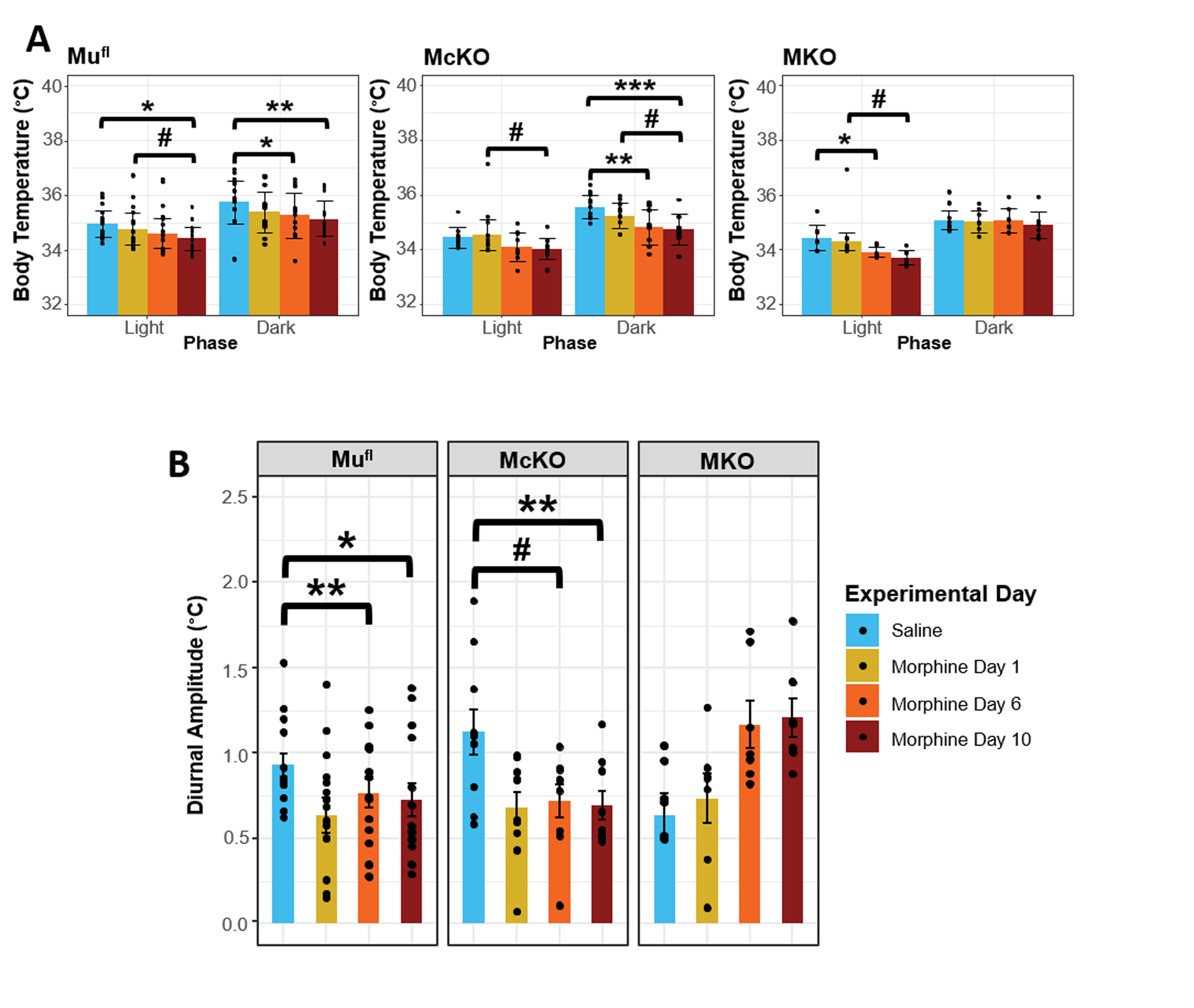

Supplement: Supplementary file 1 [file ijms-23-15870-s001.zip › FigureS4.tif]

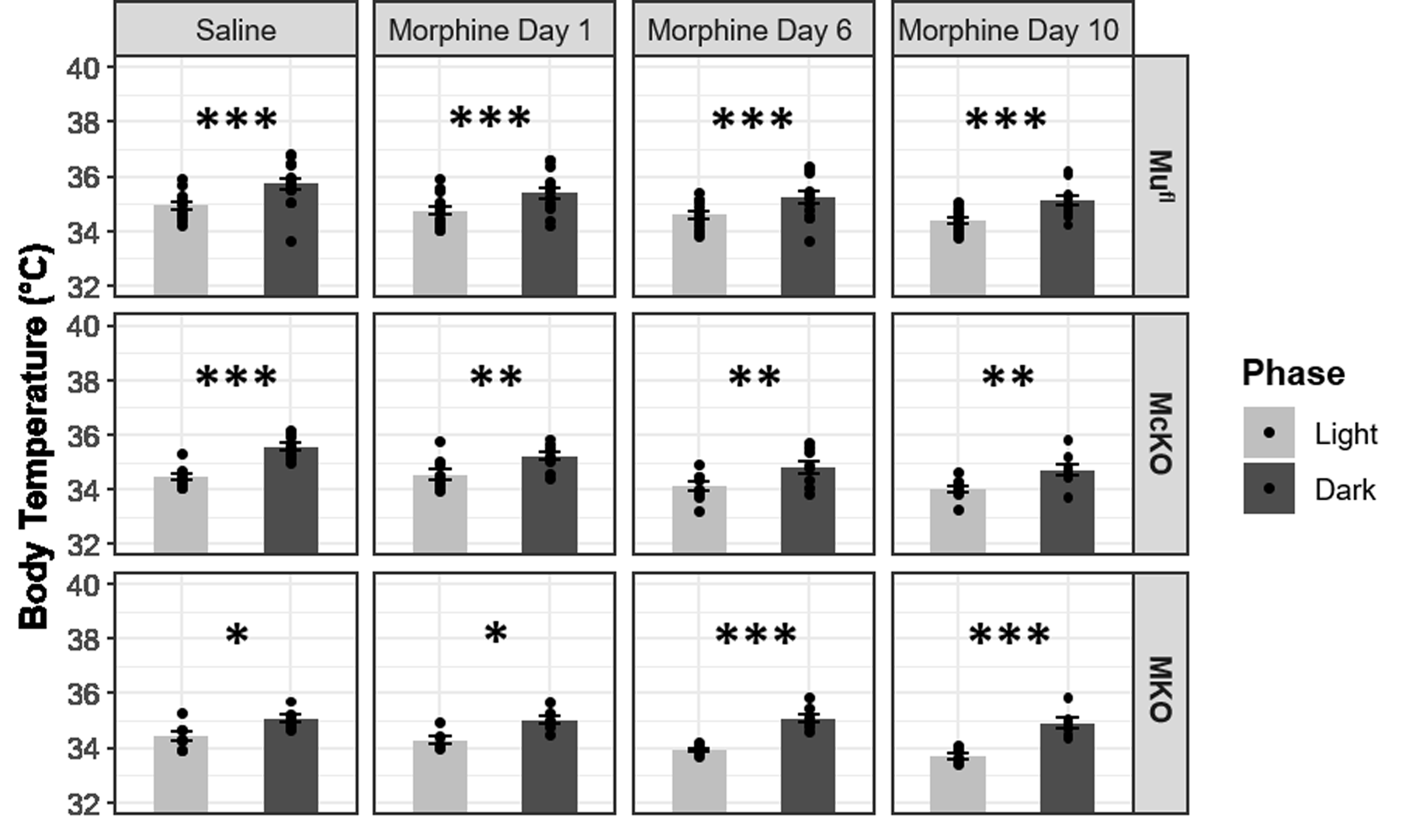

Supplement: Supplementary file 1 [file ijms-23-15870-s001.zip › FigureS5.tif]

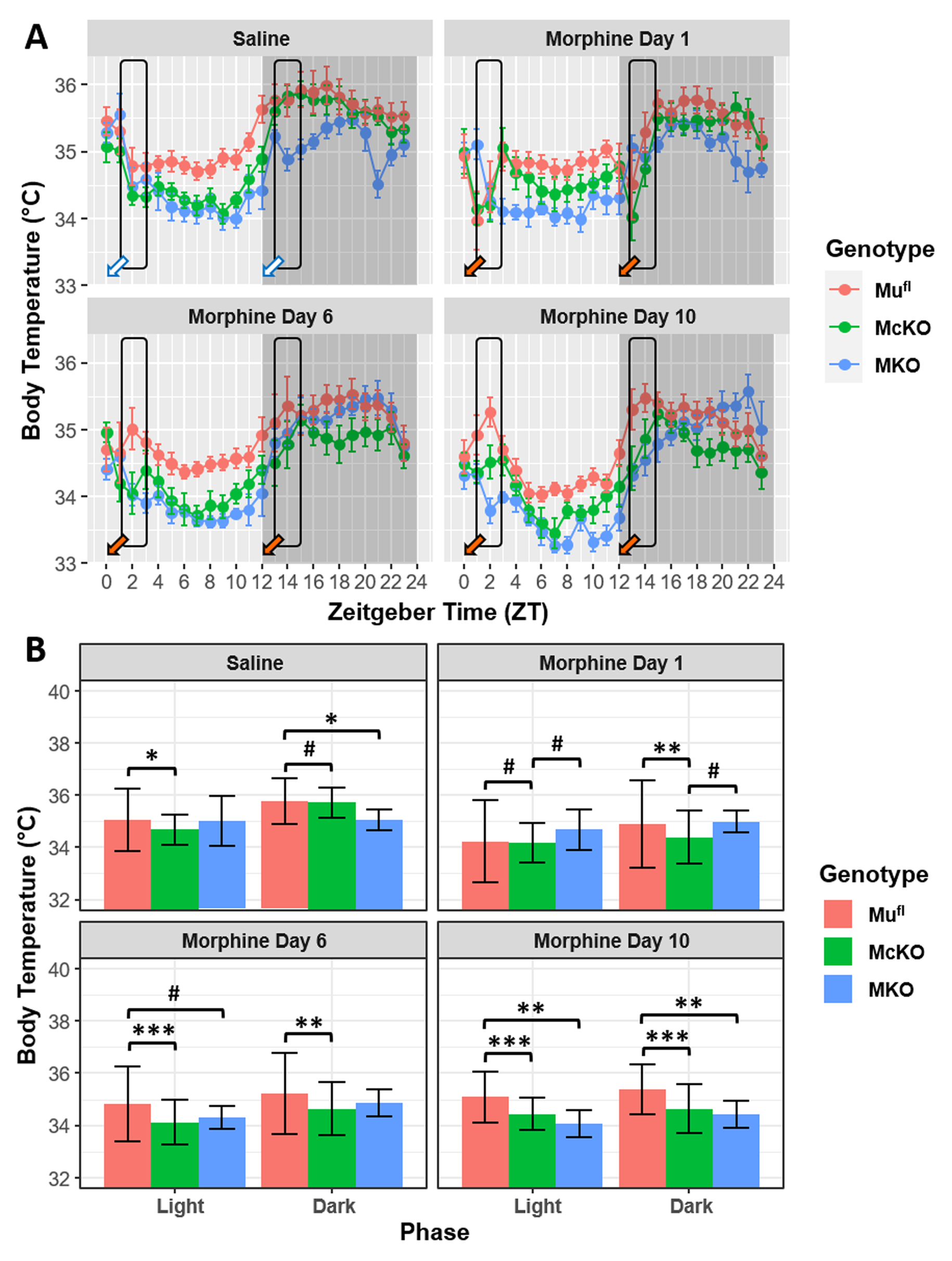

Supplement: Supplementary file 1 [file ijms-23-15870-s001.zip › FigureS6.tif]

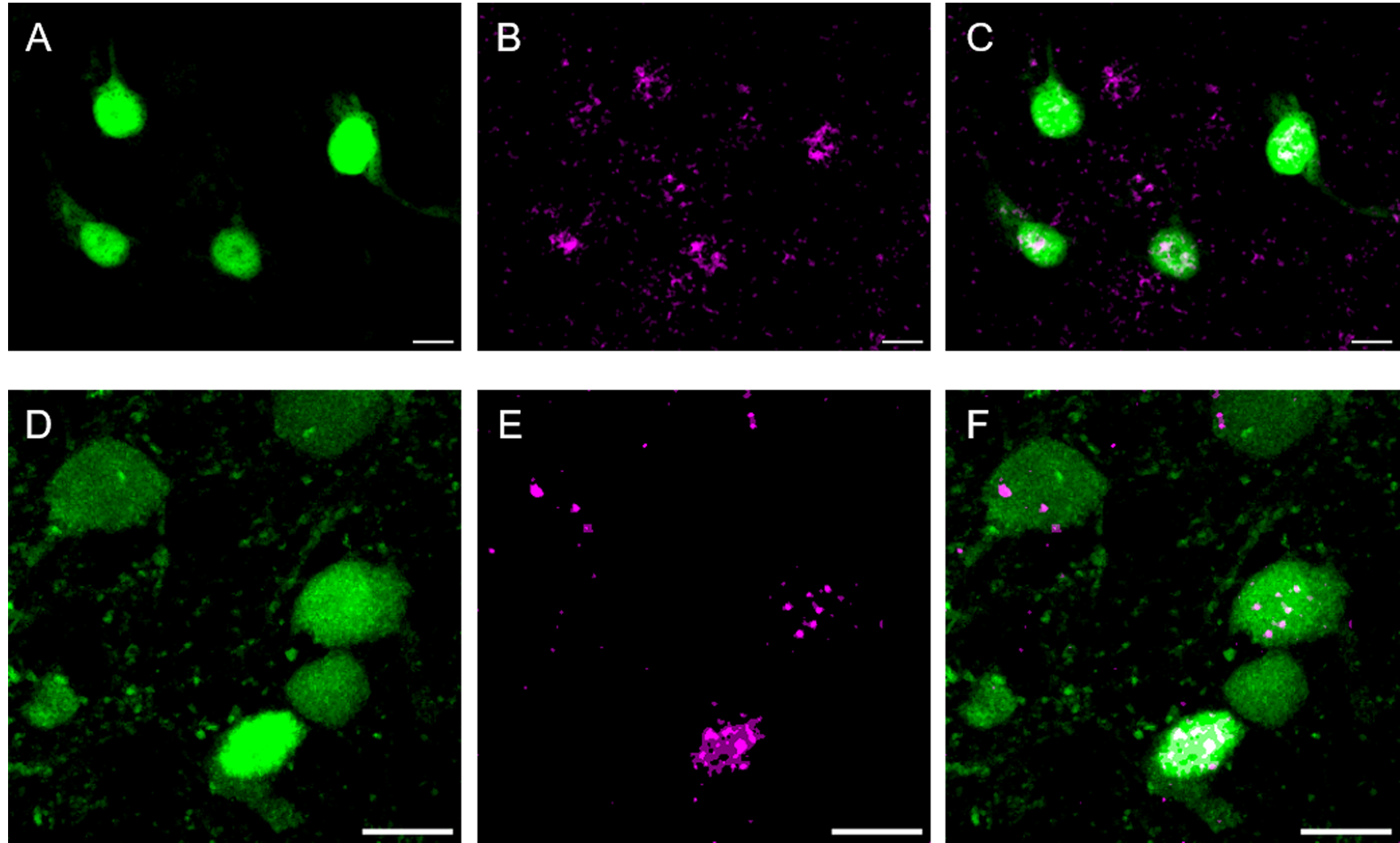

Supplement: Supplementary file 1 [file ijms-23-15870-s001.zip › FigureS7.tif]

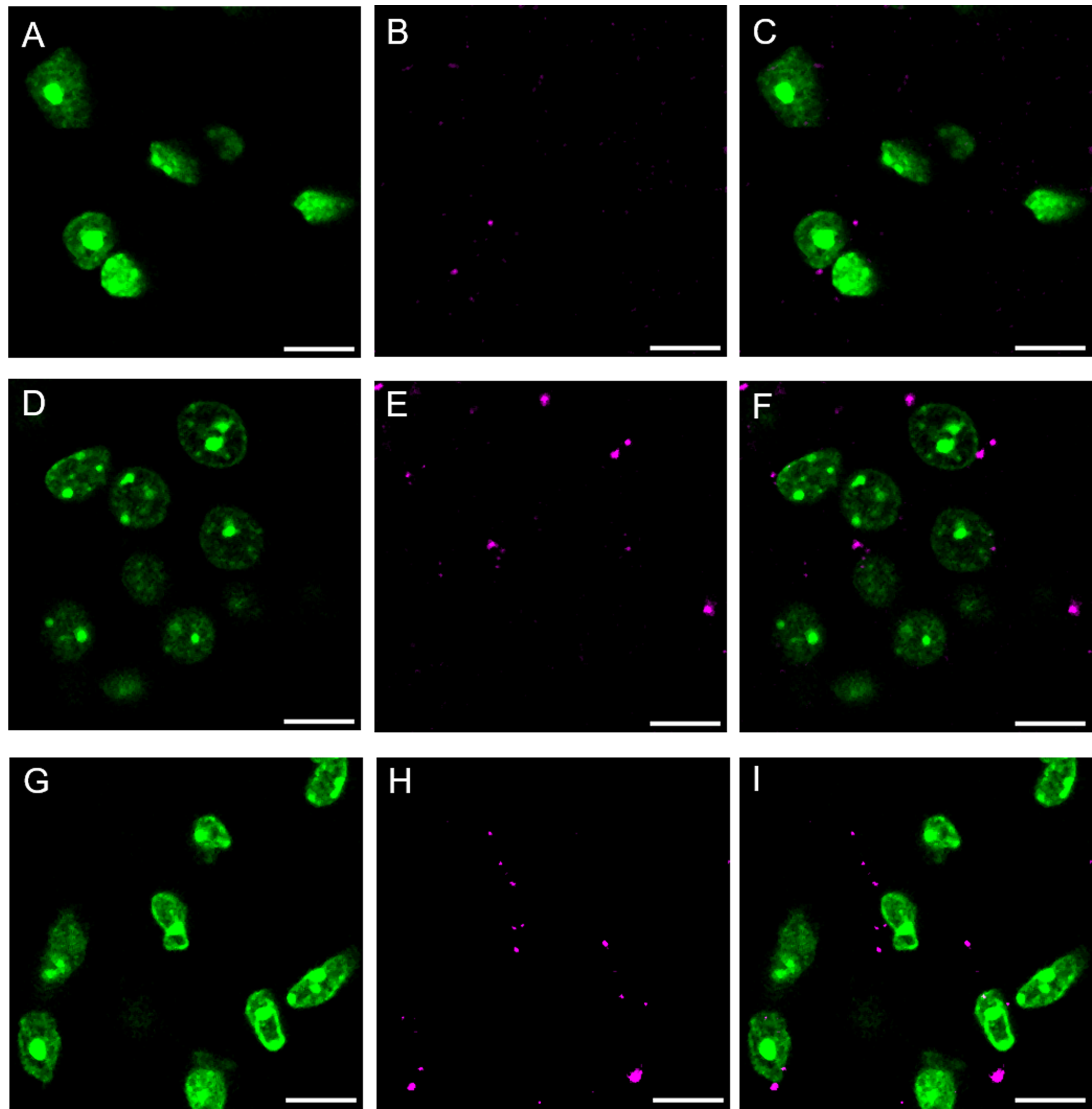

Supplement: Supplementary file 1 [file ijms-23-15870-s001.zip › FigureS8.tif]
